# Laparoscopic transabdominal approach for radical excision of low-lying presacral cyst: a case report and narrative mini−review

**DOI:** 10.3389/fonc.2026.1791445

**Published:** 2026-06-04

**Authors:** Xirang Wang, Maoxing Liu, Fei Tan, Yuxiang Li, Xiaofeng Sun

**Affiliations:** 1 Department of General Surgery, Beijing Fengtai You’anmen Hospital, Beijing, China; 2 Department of Gastrointestinal Oncology, Ward 4, Beijing Cancer Hospital, Beijing, China

**Keywords:** case report, laparoscopy, low-lying presacral cyst, radical excision, surgery

## Abstract

**Background:**

Presacral cysts are clinically rare, deeply located, and pose diagnostic challenges prior to surgery. Complete surgical resection is the primary treatment. Laparoscopic techniques offer minimally invasive advantages; however, the feasibility and safety of the transabdominal approach for low-lying cysts remain to be fully evaluated. Through this case report and narrative mini−review, this study aims to assess the technical characteristics and value of this approach.

**Clinical presentation:**

A 39-year-old female patient, with a history of constipation, no other significant medical history, and a prior cesarean section, presented due to a presacral cystic mass discovered during a routine gynecological ultrasound. She reported no specific discomfort such as abdominal distension or pain. Contrast-enhanced abdominopelvic CT revealed a cystic, low-density lesion in the presacral region (approximately 8.3 cm × 6.2 cm, with a pre-contrast CT value of about 28 HU), showing no significant enhancement. Pelvic MRI demonstrated that the cystic lesion exhibited slightly low signal on T1-weighted images (T1WI), slightly high signal on T2-weighted images (T2WI), markedly high signal on diffusion-weighted imaging (DWI), and low signal on apparent diffusion coefficient (ADC) map. The superior pole of the cyst was at the level of the coccyx. Digital rectal examination revealed a soft mass posterolateral to the rectum. After multidisciplinary discussion and thorough communication with the patient, a laparoscopic exploration was decided upon. Intraoperatively, a well-defined cystic tumor was visualized posterior to the rectum and was completely resected. Postoperative pathological diagnosis confirmed a (presacral) epidermoid cyst. At the 3-month follow-up, the patient recovered well with no recurrence or complications.

**Conclusion:**

The laparoscopic transabdominal approach appears feasible for radical excision of low−lying presacral cysts in selected patients when performed by experienced surgeons. Long−term follow−up is required to confirm recurrence outcomes.

## Introduction

Presacral cysts represent a relatively rare clinical entity, originating from embryonic remnants or congenital anomalies within the presacral space. They are predominantly benign cystic lesions, including subtypes such as epidermoid cysts and tailgut cysts (1). Due to their deep-seated location and complex anatomical relationships, often adjacent to the sacral plexus, rectum, and vascular structures, preoperative imaging diagnosis poses significant challenges, leading to potential misdiagnosis or oversight. Clinical manifestations are mostly occult, with only a minority of patients presenting with pelvic pain or mass effect (2, 3). Currently, complete surgical excision is regarded as the only effective curative method, aiming to prevent recurrence and potential malignant transformation. Although traditional open surgery can achieve complete resection, it is associated with considerable surgical trauma, prolonged postoperative recovery, and increased risk of complications (4).

Minimally invasive laparoscopic techniques have been widely applied in the field of pelvic surgery in recent years, offering advantages including reduced intraoperative blood loss, less postoperative pain, shorter hospital stays, and accelerated recovery (5, 6). However, the feasibility and safety of the transabdominal approach for low-lying presacral cysts remain subjects of debate (4). Furthermore, research on laparoscopic approaches for presacral cysts has primarily focused on high or mid-level lesions. Data on the technical feasibility and long-term outcomes for low-lying cysts are scarce, owing to factors such as limited operative space and increased anatomical complexity, resulting in a lack of consensus in clinical guidelines (7).

This study, through a case report of laparoscopic transabdominal radical excision for a low-lying presacral epidermoid cyst combined with a literature review, aims to evaluate the technical characteristics, perioperative safety, and recurrence risk associated with this approach, thereby providing evidence-based medical support for the minimally invasive management of low-lying presacral cysts.

## Case presentation

A 39-year-old female patient was admitted with a presacral mass discovered during a routine physical examination six months prior. The mass was incidentally found on a pelvic ultrasound during a gynecological check-up. The patient reported no specific discomfort such as abdominal distension or pain. Her bowel habits consisted of dry, yellow stools every 2–3 days, with no notable changes in urination. Her medical history included constipation for nearly 10 years and a previous cesarean section, with no other significant underlying conditions.

Physical examination revealed a flat abdomen with a lower abdominal surgical scar from the prior operation. The abdomen was soft throughout, with no palpable abnormal masses or hepatosplenomegaly. No shifting dullness was detected. Bowel sounds were normal at 4–5 per minute. Digital rectal examination identified a soft mass posterolateral to the rectum.

Laboratory tests, including complete blood count, biochemistry, tumor markers, coagulation profile, and infectious disease panel, showed no significant abnormalities.

Imaging studies: Contrast-enhanced abdominopelvic CT (**Figure 1**) revealed a cystic, low-density lesion in the presacral region, measuring approximately 8.3 cm × 6.2 cm with a pre-contrast CT value of about 28 HU, demonstrating no significant enhancement. Pelvic MRI (**Figure 2**) showed the cystic lesion exhibiting slightly low signal intensity on T1WI, slightly high signal on T2WI, markedly high signal on DWI, and low signal on the ADC map. Markedly high DWI signal and low ADC signal indicate restricted diffusion, consistent with a keratin−containing cyst (epidermoid cyst). Main differential diagnoses included tailgut cyst (usually multilocular, without restricted diffusion) and dermoid cyst (often containing fat). The superior pole of the cyst was at the level of the coccyx. The sagittal T2−weighted MRI (**Figure 2**) demonstrated that the superior pole of the cyst was at the level of the coccyx. According to the Chinese Expert Consensus on Standardized Diagnosis and Treatment of Presacral Cysts (2024 Edition), a low−lying presacral cyst is defined as one with its superior pole at or below the S4 level. Thus, the present case was classified as a low−lying presacral cyst. The most adherent point was at the coccygeal level. Multidisciplinary consultation (orthopedics, neurosurgery) concluded that connection to the spinal canal was unlikely. Colonoscopy revealed no significant abnormalities.

**Figure 1:**
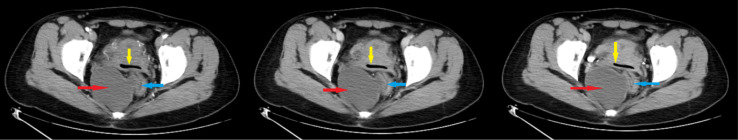
Contrast-enhanced abdominopelvic CT. Red arrow, presacral cyst; Yellow arrow, cervicovaginal region; Blue arrow, rectum.

**Figure 2:**
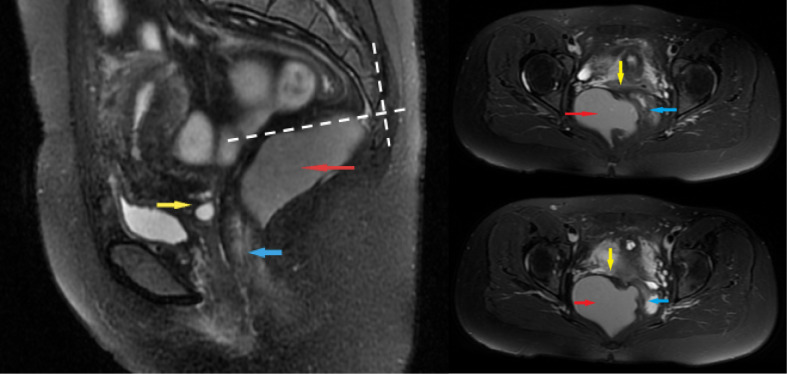
Pelvic MRI. The white dashed line indicates the method for determining the superior extent of the cyst: a perpendicular line is drawn from the highest point of the cyst to the tangent line of the adjacent sacral surface. The intersection point of the extension of this perpendicular line with the sacrum corresponds to the superior level of the cyst, which in this case is at the coccygeal level. Red arrow, presacral cyst; Yellow arrow, cervicovaginal region; Blue arrow, rectum.

### Diagnoses: presacral cyst, constipation, and status post cesarean section

Following multidisciplinary discussion and thorough communication with the patient and her family, who requested surgical removal of the presacral mass, a decision was made to proceed with laparoscopic exploration under general anesthesia. The patient was informed of potential risks of cyst enlargement, infection, or rare malignant transformation; she opted for surgical excision.

Surgical procedure: A five-port technique was employed. A 10-mm trocar was placed at the supraumbilical margin, a 12-mm trocar was placed 2 fingerbreadths medial to the right anterior superior iliac spine, 5-mm trocars were placed at the left and right midclavicular lines at the umbilical level, and an additional 5-mm trocar was placed at the left anti-McBurney’s point. Upon entering the abdominal cavity, no ascites was noted. The liver, spleen, gastrointestinal tract, omentum, and mesentery appeared smooth and free of nodules. Using an ultrasonic scalpel, the peritoneum at the junction of the sigmoid colon and the pelvic inlet was incised. Dissection proceeded along the mesorectal plane towards the anal side, revealing the presacral mass located posterolateral to the rectum. The mass was cystic, approximately 10 cm in diameter. Blunt and sharp dissection along its capsule was performed with careful preservation of the pelvic floor nerves, vasculature, and rectum. Blunt and sharp dissection along the cyst capsule was performed with careful preservation of the pelvic floor nerves, vasculature, and rectum. The bilateral hypogastric nerves and pelvic splanchnic nerves were identified and preserved. The mass was completely excised without rupture, and its capsule remained intact. The specimen was placed in a retrieval bag, shown to the family after extraction, and sent for pathological examination. The surgical field was inspected and showed no active bleeding. The total operative time was 176 minutes, with an estimated blood loss of 10 mL. No intraoperative complications or cyst rupture occurred. After irrigation, the pelvic floor peritoneum was closed with absorbable sutures to prevent small bowel herniation or adhesion formation (as shown in **Figure 3**). To prevent localized fluid collection from oozing or exudation, one drain was placed in the presacral space (beneath the sutured peritoneum) and another drain was placed in the pelvic cavity (above the peritoneum). The drains were removed when the output decreased to less than 50 mL per day with clear serous fluid. The patient tolerated oral intake well on postoperative day 1. The pelvic drain was removed on postoperative day 2 and the presacral drain on postoperative day 3. She was discharged on postoperative day 4 without any complications (Clavien−Dindo grade 0).

**Figure 3:**
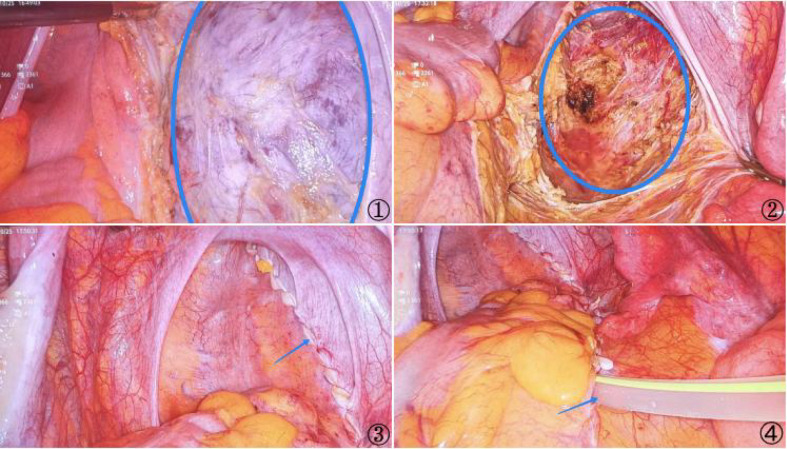
Intraoperative findings and post-resection management. ① The outlined area demarcates the tumor after incision of the mesenteric serosal layer, revealing its intraoperative contour; ② The outlined area shows the surgical bed after complete tumor resection; ③ The blue arrow indicates the view after suturing the serosal layer over the surgical field; ④ The blue arrow points to a drainage tube placed in the presacral surgical bed beneath the sutured serosal layer.

The extracted specimen is shown in **Figure 4**. Postoperative pathological examination confirmed the diagnosis of a (presacral) epidermoid cyst. High−resolution HE−stained photomicrographs (**Figure 5**) showed the cyst wall lined by stratified squamous epithelium, with no evidence of malignancy or teratomatous components. At the 3-month follow-up, she recovered well. Imaging studies showed no evidence of fluid collection or recurrence, and her constipation had improved. She reported no new−onset voiding or sexual dysfunction.

**Figure 4:**
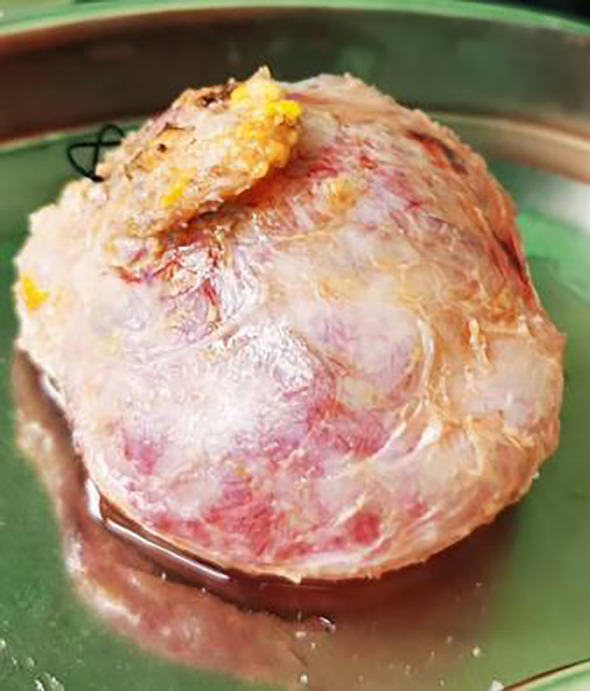
Resected specimen with an intact, unruptured cystic capsule.

**Figure 5:**
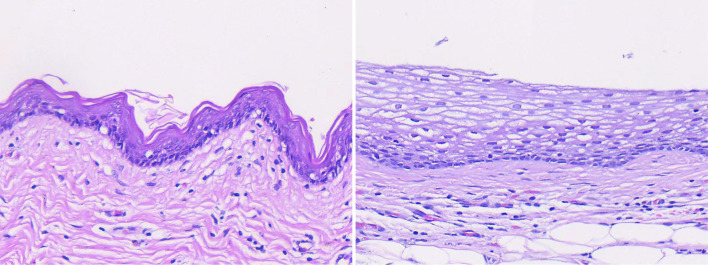
Histopathology (HE staining). High-power view showing cyst wall lined by stratified squamous epithelium and confirming no malignancy or teratomatous components.

## Discussion and conclusions

A presacral epidermoid cyst is a rare benign neoplastic condition, primarily composed of epidermal tissue, which can cause local compressive symptoms such as pain, or difficulties with urination or defecation. Due to its deep-seated location and proximity to critical anatomical structures (e.g., the sacral nerve plexus and rectum), surgical excision is the primary treatment, necessitating meticulous technique to avoid complications such as nerve injury or cyst rupture (8). In the present case, the mass was low-lying, and a transabdominal laparoscopic resection was successfully performed without postoperative complications, supporting the feasibility of this approach in selected patients. Rationale for the anterior laparoscopic approach: The anterior (transabdominal) laparoscopic approach was chosen over posterior/perineal or combined approaches for the following reasons: (1) it provides superior visualization of the rectum and pelvic autonomic nerves (hypogastric nerves and pelvic splanchnic nerves), facilitating complete resection with nerve preservation; (2) it avoids a perineal incision, thereby reducing the risk of wound infection, sinus formation, and postoperative pain; (3) it allows en bloc resection while maintaining pelvic floor integrity; (4) in low−lying cysts (below S4), the transabdominal approach still enables adequate access by mobilizing the rectum and dissecting along the mesorectal plane. These considerations are consistent with the 2024 Chinese Expert Consensus (9). Notably, cyst rupture is a primary risk in laparoscopic surgery; however, literature indicates that even if rupture occurs, the incidence of severe complications such as chemical peritonitis is very low (10).

Robotic−assisted resection of presacral cysts has been reported in small series, offering better dexterity in the deep pelvis. However, laparoscopy remains more cost−effective and widely available. In this case, laparoscopy achieved complete resection without robotic assistance. We employed a laparoscopic transabdominal approach, emphasizing comprehensive preoperative evaluation (including imaging to localize the cyst and exclude malignancy) and meticulous intraoperative technique. The patient recovered uneventfully postoperatively, with no adverse events such as nerve injury or infection, highlighting the applicability of laparoscopic surgery in the low presacral region. In conjunction with the literature, the success of laparoscopic surgery relies on surgeon experience, appropriate instrument selection (e.g., ultrasonic scalpel, suction systems to prevent spillage of cyst contents), and multidisciplinary collaboration to address the anatomical complexity of the presacral space (11). This case also illustrates the importance of individualized decision-making: selecting laparoscopy over other approaches based on cyst size, location, and patient symptoms can maximize clinical benefit.

The follow−up period is only 3 months, which is insufficient to assess recurrence. Long−term imaging surveillance is necessary, and this remains a major limitation of this report. In conclusion, although presacral epidermoid cysts are rare, surgical resection is crucial for cure. The successful execution in this case confirms that, with strict preoperative assessment and precise intraoperative technique, laparoscopic transabdominal radical excision for low−lying presacral cysts is feasible in selected patients when performed by experienced surgeons. Long−term follow−up is required to confirm recurrence outcomes. Multicenter studies with longer follow−up are warranted to evaluate long−term efficacy and recurrence rates.

## Data Availability

The raw data supporting the conclusions of this article will be made available by the authors, without undue reservation.

## References

[1] Perrone E Shaheen S Kunz PL Henri HC Baum RP . First autopsy-confirmed complete remission of metastatic neuroendocrine neoplasm of tailgut cyst after a single cycle of alpha-peptide receptor radionuclide therapy with [225Ac]Ac-DOTA-LM3. Clin Nucl Med. doi: 10.1097/rlu.0000000000006201. PMID: 41308086

[2] Stelzner S Heinze T Heimke M . Beyond total mesorectal excision: compartment-based anatomy of the pelvis revisited for exenterative pelvic surgery. Ann Surg. (2023) 278:e58–67. 10.1097/SLA.0000000000005715 36538640

[3] Medvediev M Spesyvtsev D Pokrovenko D . A case of neovagina surgical creation using the uterine cervix remnant in a patient with Mayer-Rokitansky-Küster-Hauser syndrome. Fertil Steril. (2021) 116:1420–2. doi: 10.1016/j.fertnstert.2021.06.030. PMID: 34330430

[4] Kuehn R Uchiumi LJ Tamarozzi F . Treatment of uncomplicated hepatic cystic echinococcosis (hydatid disease). Cochrane Database Syst Rev. (2024) 7:CD015573. doi: 10.1002/14651858.cd015573. PMID: 38994714 PMC11240857

[5] Brimo Alsaman MZ Ali SM Kitaz MN . Comprehensive review: types, clinical manifestations, diagnosis, and surgical management of ectopic gallbladder. Int J Surg. doi: 10.1097/js9.0000000000003332. PMID: 40990476 PMC12825902

[6] Corbett GA Windrim C Higgins S . Laparoscopic prepregnancy transabdominal cerclage: operative and pregnancy outcomes for a novel technique of suture placement using port closure device. Am J Obstet Gynecol. (2024) 230:578–80. doi: 10.1016/j.ajog.2023.12.002. PMID: 38065377

[7] Roman H Merlot B Darwish B . Excision of deep endometriosis nodules of the parametrium and sacral roots in 10 steps. Fertil Steril. (2021) 115:1586–8. doi: 10.1016/j.fertnstert.2021.02.014. PMID: 33766459

[8] Quak E Catroux A Lasne-Cardon A Ciappuccini R . False-positive skin uptake on 18F-choline PET/CT related to an inflammatory epidermoid cyst in a patient with parathyroid adenoma. Clin Nucl Med. doi: 10.1097/rlu.0000000000006291. PMID: 41428746

[9] Pelvic Tumor Integration Committee of China Anti-Cancer Association Pelvic Oncology Branch of China International Exchange And Promotive Association For Medical And Healthcare Digestive Tract Polyps and Precancerous Lesions Committee of China Anti-Cancer Association Combined Organ Excision and Quality Control Group, Colorectal Cancer Committee of Chinese Medical Doctor Association . Zhonghua Zhong Liu Za Zhi. (2025) 47:76–85. 39632258

[10] Tankou J Foley OW Liu CY Melamed A Schantz-Dunn J . Dermoid cyst management and outcomes: a review of over 1000 cases at a single institution. Am J Obstet Gynecol. (2024) 231:442.e1–7. doi: 10.1097/01.ogx.0001110360.02152.be. PMID: 38670445

[11] Orlando MS Carey-Love A Attaran M King CR . Surgical techniques for excision of juvenile cystic adenomyoma. Fertil Steril. (2022) 118:810–1. doi: 10.1016/j.ajog.2021.12.234. PMID: 35931491

